# Supplementary material for: Hydrolytic vs. Nonhydrolytic Sol-Gel in Preparation of Mixed Oxide Silica–Alumina Catalysts for Esterification
Source: Molecules. 2022 Apr 14;27(8):2534. doi: 10.3390/molecules27082534 (PMC9029862; doi:10.3390/molecules27082534)
Supplement: Supplementary file 1 [file molecules-27-02534-s001.zip › molecules-1674486-supplementary.pdf]

SUPPLEMENTARY MATERIALS

Hydrolytic vs. Nonhydrolytic Sol-Gel in Preparation of Mixed Oxide Silica–Alumina Catalysts for Esterification

Atheer Al khudhair [1,2,*], Karim Bouchmella [1], Pierre Hubert Mutin [1], Vasile Hulea [1], Olinda Gimello [1] and Ahmad Mehdi [1,*]

[1] ICGM, University Montpellier, CNRS, ENSCM, 34095, Montpellier, France; karim.bouchmella@umontpellier.fr (K.B.); hubert.mutin@umontpellier.fr (P.H.M.); vasile.hulea@umontpellier.fr (V.H.); olinda.gimelle@umontpellier.fr (O.G.)
[2] Department of Chemistry, College of Science, University of Kerbala, Karbala 56001, Iraq
* Correspondence: atheer.h@uokerbala.edu.iq (A.A.); ahmad.mehdi@umontpellier.fr (A.M.)

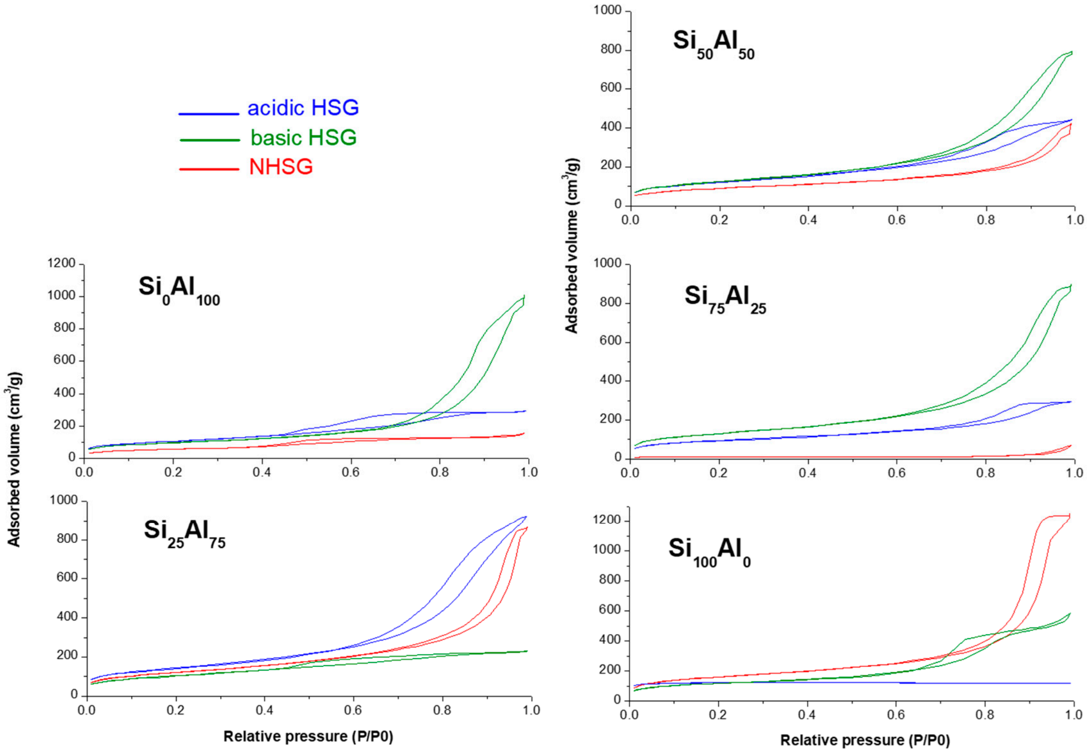

Figure S1. N_2 adsorption–desorption isotherms of Si_xAl_y samples.

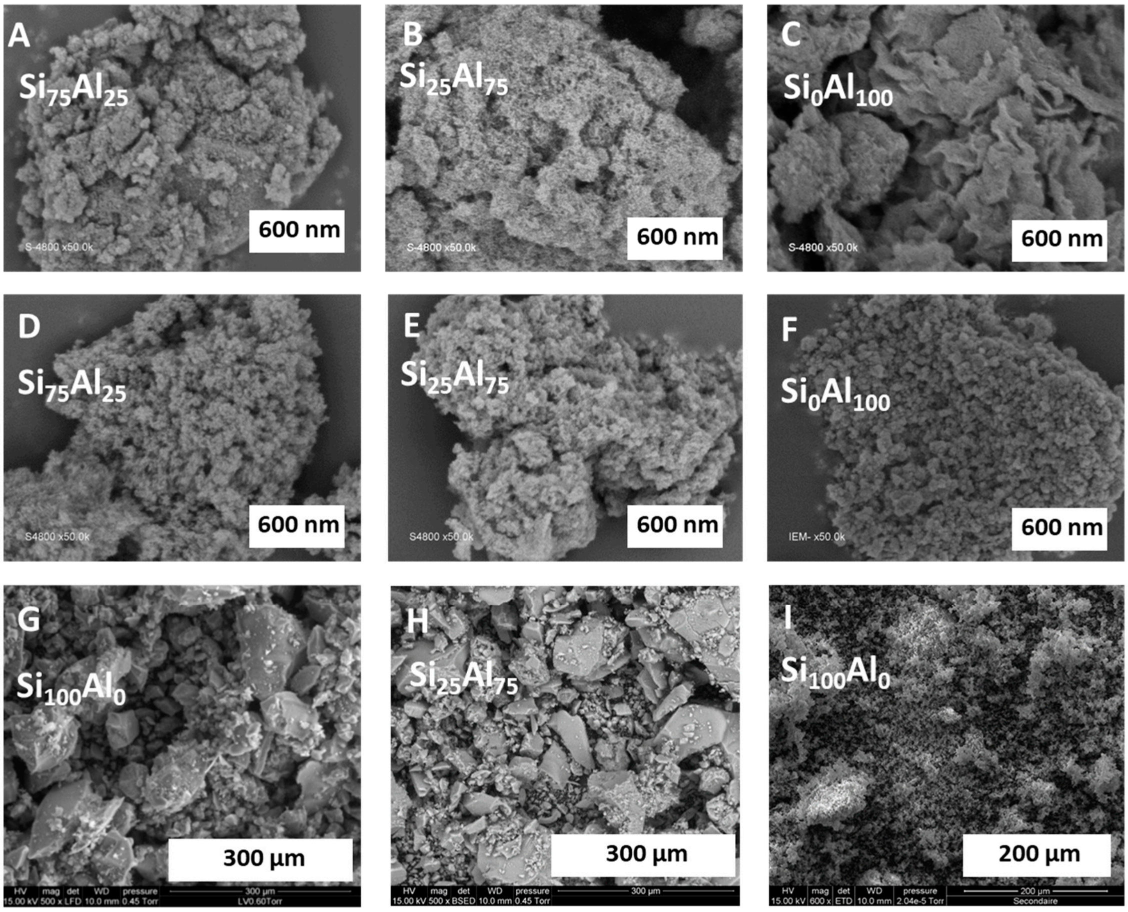

Figure S2: SEM images of samples prepared by acidic HSG (**A**, **B**, **C**), basic HSG (**D**, **E**, **F**) and NHSG (**G**, **H**, **I**).

Table S1: XRD data of γ-Al_2O_3

2θ (deg)	hkl, ICDD: 29-1486, 29-0063, 10-0425
19.58	[111]
34.83	[220]
37.38	[311]
39.40	[222]
41.26	
45.73	[400]
60.85	[511]
66.85	[440]
84.87	[444]
